# Case report: *Mafb* promoter activity may define the alveolar macrophage dichotomy

**DOI:** 10.3389/fimmu.2022.1050494

**Published:** 2022-12-12

**Authors:** Thao Vo, Yogesh Saini

**Affiliations:** Department of Comparative Biomedical Sciences, School of Veterinary Medicine, Louisiana State University, Baton Rouge, LA, United States

**Keywords:** MAFB, Cre-LoxP, macrophage-specific Cre, alveolar macrophages, lung

## Abstract

Cre-LoxP system has been widely used to induce recombination of floxed genes of interest. Currently available macrophage promoter-specific Cre recombinase mice strains have various limitations that warrants the testing of additional Cre strains. V-maf **m** usculo **a** poneurotic **f** ibrosarcoma oncogene family, protein **b** -*Cre* (*Mafb-Cre*) mice label macrophages in most organs such as spleen, small intestine, lung, bone marrow, and peritoneal cavity. However, whether *Mafb-Cre* recombinase targets the gene recombination in alveolar macrophage remains untested. Here, we utilized *Mafb^Cre/WT^R26^mTmG/WT^* strain that expresses mTOM protein in all the cells of mouse body except for those that express *Mafb*-Cre-regulated mEGFP. We performed fluorescent microscopy and flow cytometry to analyze mTOM and mEGFP expression in alveolar macrophages from *Mafb^Cre/WT^R26^mTmG/WT^* mice. Our analyses revealed that the *Mafb*-Cre is active in only ~40% of the alveolar macrophages in an age-independent manner. While *Mafb*- (mTOM+/mEGFP-) and *Mafb*+ (mEGFP+) alveolar macrophages exhibit comparable expression of CD11b and CD11c surface markers, the surface expression of MHCII is elevated in the *Mafb*+ (mEGFP+) macrophages. The bone marrow-derived macrophages from *Mafb^Cre/WT^R26^mTmG/WT^* mice are highly amenable to Cre-LoxP recombination *in vitro.* The bone marrow depletion and reconstitution experiment revealed that ~98% of alveolar macrophages from *Mafb^Cre/WT^R26^mTmG/WT^* → WT chimera are amenable to the *Mafb*-Cre-mediated recombination. Finally, the *Th2* stimulation and ozone exposure to the *Mafb^Cre/WT^R26^mTmG/WT^* mice promote the *Mafb*-Cre-mediated recombination in alveolar macrophages. In conclusion, while the *Mafb*-/*Mafb*+ dichotomy thwarts the use of *Mafb-Cre* for the induction of floxed alleles in the entire alveolar macrophage population, this strain provides a unique tool to induce gene deletion in alveolar macrophages that encounter *Th2* microenvironment in the lung airspaces.

## Introduction

Cre-LoxP system has been widely used to induce recombination of floxed genes of interest (1, 2). In this system, the Cre recombinase enzyme recognizes two target 34bp LoxP (locus of x-over, P1) unidirectional sequences in genomic DNA and catalyzes a recombination reaction where floxed allelic region, i.e., a sequence that is flanked by two LoxP sequences, is excised (3). The gene recombination can be restricted to a particular cell type by employing a cell type-specific promoter to induce Cre recombinase transgene expression in a cell type-specific manner. Numerous mouse strains with Cre recombinase expression in single cell type including hepatocytes (4), alveolar type II cells (5, 6), club cells (7), myocardium (8) are available. Due to the relatively higher degree of plasticity in immune cells (9), including macrophages (10), widely acceptable immune cell-specific Cre recombinase mouse strains are very limited.

Several Cre recombinase mouse strains utilizing myeloid-associated promoters such as *Lysozyme M* (11–14), Colony Stimulating Factor 1 Receptor (*Csf1r)* (15)*, CD11b* (16–18), *CD11c* (19–22), C-X3-C Motif Chemokine receptor 1 (*Cx3cr1)* (23), and *F4/80* (24) have been used to explore macrophage-specific roles. These strains, however, are known to target non-macrophage cell types as well (14, 23–32). Therefore, mouse strains that restrict Cre recombinase specifically to the macrophages are still awaited. V-maf **m** usculo **a** poneurotic **f** ibrosarcoma oncogene family, protein **b** (*Mafb*) promoter has been reported to express in macrophages (33). In that report, *Mafb*-Cre mouse strain was employed to demonstrate that there was a clear separation of macrophage and dendritic cell (DC) populations based on the expression of MAFB and ZBTB46, respectively (33). Moreover, *Mafb*-Cre mice appeared to be a reliable alternative to trace and distinguish macrophages from other cell types, especially DCs, in most organs such as spleen, small intestine, lung, bone marrow, and peritoneal cavity (33). However, the capability of *Mafb-cre* recombinase in inducing floxed gene recombination in alveolar macrophage remains untested.

In the current study, we sought to determine the effectiveness of *Mafb*-regulated Cre recombinase in the induction of recombination within a floxed mTOM/mEGFP (mTmG) reporter allele. Therefore, we utilized *R26^mTmG/mTmG^* reporter strain that expresses mTOM protein in all the cells of mouse body except for those that express Cre recombinase enzyme (34). The Cre recombinase-expressing cells in *R26^mTmG/mTmG^* reporter mice translate mEGFP protein instead of mTOM, a readout for a successful recombination event. We hypothesized that all the alveolar macrophages from the *Mafb^Cre/WT^R26^mTmG/WT^* mice express mEGFP protein. To address this hypothesis, we harvested alveolar macrophages from *Mafb^Cre/WT^R26^mTmG/WT^* mice and analyzed their mTOM and mEGFP expression status using fluorescent microscopy and flow cytometry. Our data suggest that *Mafb* gene determines the alveolar macrophage dichotomy, which is independent of the recruitment of bone marrow-derived macrophages.

## Materials and methods

### Generation of *Mafb^Cre/WT^R26^mTmG/WT^* mice and animal husbandry

*Mafb*-regulated Cre recombinase expressing line (B6N(129S4)-*Mafb^tm1.1(cre)Kmm^*/J), Rosa promoter (R26) regulated mTOM/mEGFP (mTmG) dual fluorescent reporter line (B6.129(Cg)-*Gt26Sor^tm4(ACTB-tdTomato,-EGFP)Luo^*/J) were procured from Jackson Laboratory (Bar Harbor, ME). *Mafb^Cre/WT^* mice had mixed C57BL/6J and C57BL/6N background while mTOM/mEGFP reporter mice were from C57BL/6J background. These two strains were crossed to generate *Mafb^Cre/WT^R26^mTmG/WT^* mice. All mice used in this study were maintained in hot-washed, individual ventilated cages, strictly followed 12-hour dark/light cycle and were fed regular diet with water *ad libitum*. All animal procedures were performed under animal protocol approved by the Institutional Animal Care and Use Committee (IACUC) of the Louisiana State University.

### Bronchoalveolar lavage fluid collection

*Mafb^Cre/WT^R26^mTmG/WT^* neonates (PND 3) and adults (PND 42) were anesthetized *via* intraperitoneal injection (11) of 2,2,2-tribromoethanol (Millipore Sigma, Burlington, MA). After midline laparotomy, lung and trachea were exposed *via* thoracotomy. Whole lung was lavaged with phosphate buffered saline (PBS) and processed, as previously described (35). BAL cells were processed for flow cytometry and fluorescent microscopic analyses.

### Flow cytometry

BAL cells were Fc-blocked with CD16/32 (Thermo Fisher Scientific, Waltham, MA) and stained with leukocyte panel, including AF700 CD45 (BioLegend, San Diego, CA), BV510 CD11c (Thermo Fisher Scientific, Waltham, MA), BV785 CD11b (BioLegend, San Diego, CA), BV605 Ly6G (BioLegend, San Diego, CA), BV421 CD64 (BioLegend, San Diego, CA), APC CD24 (BioLegend, San Diego, CA), and PeCP-Cy5.5 MHCII (BioLegend, San Diego, CA) to characterize the alveolar macrophage population, which was further analyzed for mTOM+ and mEGFP+ subpopulations using Cytoflex (Beckman Coulter, Inc., CA). Flow cytometry data was analyzed by CytExpert Software (Beckman Coulter, Inc., CA).

### Oropharyngeal M1/M2 challenge

*Mafb^Cre/WT^R26^mTmG/WT^* mice were anesthetized with isoflurane and oropharyngeal challenged with a cocktail of *Th1* stimulants LPS [10 μl (10 μg) LPS + 40 μl saline] and IFN-γ, or *Th2* stimulant IL-33 (1.25 μg IL-33 in 50 μl saline) for alternative (M2) activation. For M1 activation, mice were challenged with 10 μg of LPS on day 1 and the IFN-γ was instilled on day 7, followed by BALF collection of day 8. For M2 activation, mice were oropharyngeal challenged with 1.25 μg of IL-33 on days 1, 3, 5, 7 and BALF was collected on day 8.

### Ozone exposure

Ozone exposure procedure has been previously established (36). Ozone was generated from the Ozone Generator (TSE, Chesterfield, MO) and was supplied to 1.3m^3^ glass chambers. Briefly, mice were transferred into cages with perforated lids and were placed inside the dark chambers without feed and water before the start of DLAM night cycle. Ozone concentration was maintained at ~ 800 ppb throughout the 4-hour duration of the exposure. Ozone concentration along with chamber temperature, pressure and humidity were monitored and recorded at hourly interval during exposures. The timing of exposures was maintained strictly throughout the 14-day exposure.

### Bone marrow transplantation

8-10-week-old WT mice on C57BL/6J background were lethally irradiated with 6 Megavolt X-rays from a Linear Accelerator (Varian Clinac 21EX) with two (dorsal and ventral) 525-rad (525 cGy) doses (37). Femur and tibia bones of donor *Mafb^Cre/WT^R26^mTmG/WT^* mice were collected to prepare single suspension of bone marrow cells for transplantation. A total of 8 × 10^6^ cells were injected into the tail vein of lethally irradiated recipient mice. After receiving bone marrow cells *via* tail vein injection, the recipient mice were given 0.2% neomycin sulfate dissolved in acidified water for the first 2 weeks post-transplantation to reduce the bacterial growth in water bottles due to regurgitated food. Necropsies were performed 5 weeks post-bone marrow injections.

### Statistics

Student’s *t* test was used for two-group comparisons. One-way ANOVA was used for three-group comparisons. The *p* values <0.05 were considered statistically significant. All data represented at least three different experiments. GraphPad Prism 8.0 (La Jolla, CA) was used for statistical analyses. The values were represented as mean ± SEM.

## Results

### Steady-state alveolar macrophages exist as *Mafb+ and Mafb-* mixed populations

**Figure 1:**
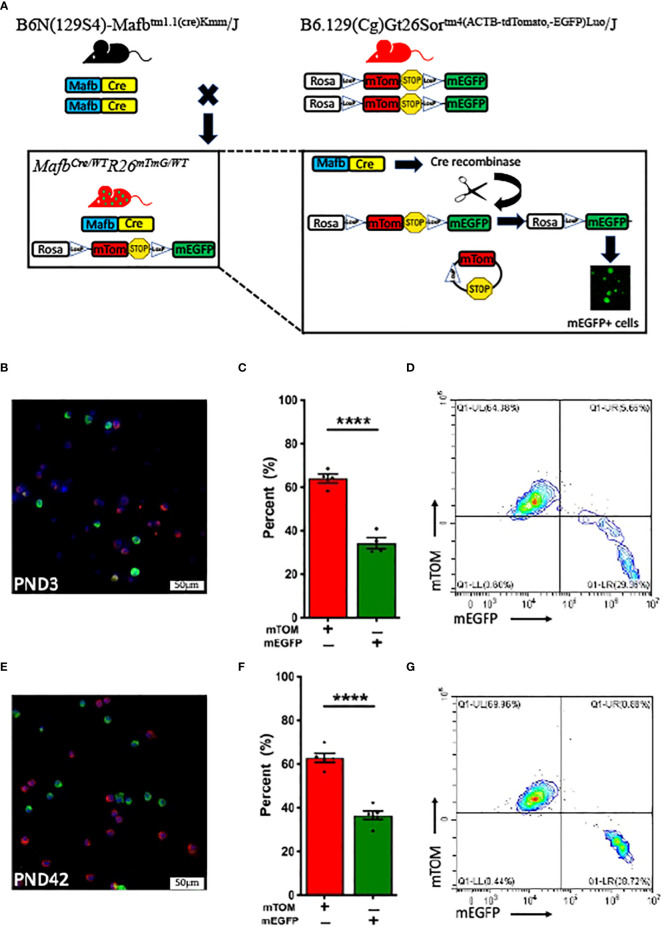
*Mafb^Cre/WT^R26^mTmG/WT^* mice at various stages of postnatal development exhibit comparable Cre-LoxP efficiency. **(A)** Generation of transgenic *Mafb^Cre/WT^R26^mTmG/WT^* mice. *Mafb*-regulated Cre recombinase expressing strain (B6N(129S4)-*Mafb^tm1.1(cre)Kmm^*/J) was crossed with Rosa promoter (R26)-regulated dual fluorescent mTOM/mEGFP reporter strain (B6.129(Cg)-*Gt26Sor^tm4(ACTB-tdTomato,-EGFP)Luo^*/J) to generate *Mafb^Cre/WT^R26^mTmG/WT^* mice. In cells with active *Mafb* promoter in *Mafb^Cre/WT^R26^mTmG/WT^* mice, Cre recombinase excises the mTOM and PolyA Stop sequences, which are flanked by the LoxP sites, and allows the translation of mEGFP protein. Representative fluorescent photomicrographs of **(B)** PND 3 (n=4) and **(E)** PND 42 (6-week-old) (n=5) BALF cells from *Mafb^Cre/WT^R26^mTmG/WT^* mice depicting the fluorescent cell composition and respective percentage of exclusively mTOM+ and mEGFP+ cells in **(C)** PND 3 and **(F)** PND 42 *Mafb^Cre/WT^R26^mTmG/WT^* mice. Error bars represent SEM ****p<0.0001 using Student’s *t* test. Representative flow cytometry graphs depicting exclusively mTOM+ (UL) and mEGFP+ (UR+LR) cells, respectively in BALF from **(D)** PND 3 and **(G)** PND 42 *Mafb^Cre/WT^R26^mTmG/WT^* mice.

We crossed *Mafb^Cre/WT^* and *R26^mTmG/mTmG^* reporter mice to generate *Mafb^Cre/WT^R26^mTmG/WT^* strain (**Figure 1A**). First, we analyzed alveolar macrophages in 3-day-old *Mafb^Cre/WT^R26^mTmG/WT^* neonates. To our surprise, only ~40% (34 ± 2.4%) of the harvested alveolar macrophages expressed mEGFP green fluorescent protein, indicating *Mafb* promoter activity and associated Cre-LoxP recombination. The remaining ~60% (64 ± 2.1%) of alveolar macrophages exclusively exhibited mTOM expression suggesting the absence of *Mafb* activity and therefore the lack of Cre recombinase expression and recombination in the floxed reporter allele (**Figures 1B–D**). Because macrophages from different origins colonize the lung in three successive waves throughout the embryonic developmental stages and spatially distribute during the first week of postnatal life (38), we hypothesized that, as compared to the neonatal alveolar macrophage population, the alveolar macrophage population collected from adult *Mafb^Cre/WT^R26^mTmG/WT^* mice possesses different composition of mTOM+ and mEGFP+ macrophages. Therefore, we analyzed alveolar macrophages from 6-week-old *Mafb^Cre/WT^R26^mTmG/WT^* mice. Again, to our surprise, the adult mice also had ~40% (36.6 ± 2%) and ~60% (62.9 ± 2.1%) alveolar macrophages that exhibited mEGFP and exclusive mTOM expression, respectively (**Figures 1E–G**). These data suggest that the effectiveness of *Mafb*-regulated Cre recombination is comparable in the neonatal and matured stage of the lung, and that *Mafb* promoter is not expressed in all the steady-state alveolar macrophages.

Next, to test the hypothesis that the mTOM+ alveolar macrophages reflect immature macrophages and have the potential to express *Mafb*-regulated Cre and mEGFP expression at maturity, we harvested alveolar macrophages and plated them in a cell culture dish. During the observation period, we did not observe mEGFP positivity in the mTOM+ cells (**Supplemental Figure 1**). Consistent with this finding, the flow cytometry analyses did not reveal double positive cells (**Figure 1G**), i.e., transitional population that reflect the read out from pre-recombination (mTOM expression) as well as post-recombination (mEGFP expression) events. These data suggest a coexistence of exclusively mEGFP- and mTOM-expressing macrophage populations in the lung airspace at homeostasis.

**Figure 2:**
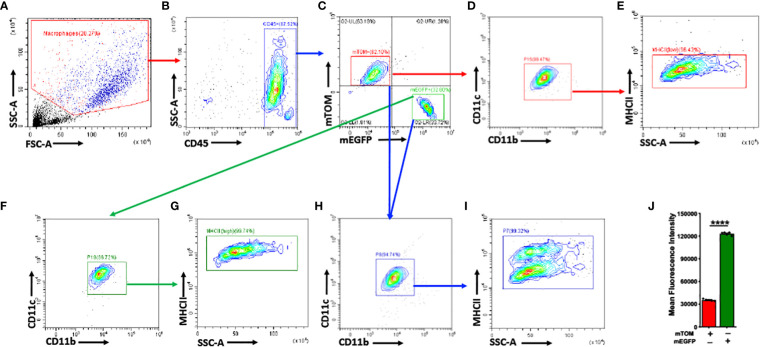
mTOM+ and mEGFP+ subpopulations share comparable myeloid surface markers, except for MHCII, in naïve *Mafb^Cre/WT^R26^mTmG/WT^*. **(A)** Applied Side Scatter (SSC) and Forward Scatter (FSC) to eliminate dead cells and debris. **(B)** Leukocyte CD45+ cells were identified. **(C)** mTOM+ and mEGFP+ subpopulations were isolated. Myeloid markers such as CD11c, CD11b, MHCII, were assessed in **(D, E)** mTOM+ and **(F, G)** mEGFP+ subpopulations. **(H)** Overlapping mTOM+ and mEGFP+ cells revealed comparable expression of CD11c and CD11b between these subpopulations. **(I)** mEGFP+ cells exhibited higher MHCII expression as compared to mTOM+ cells. **(J)** Histogram depicting mean fluorescence intensity (MFI) of MHCII expression in mTOM+ and mEGFP+ populations. Error bars represent SEM ****p<0.0001 using Student’s *t* test. Data shown are from PND 42 naïve *Mafb^Cre/WT^R26^mTmG/WT^* mice (n=5).

Further, we analyzed mTOM+ and mEGFP+ macrophages for the expression of selected myeloid cell surface markers such as CD11b, CD11c, and MHCII (**Figure 2**). Interestingly, the expression of these surface markers, except for MHCII, were comparable between the analyzed mTOM+ and mEGFP+ macrophages (**Figure 2**). mEGFP+ macrophages exhibited higher MHCII expression as compared to mTOM+ counterparts (**Figure 2J**). These data indicate a coexistence of alveolar macrophage subpopulations that display distinct MHCII expression, indicating their different antigen presentation potential.

### Bone marrow-derived macrophages are amenable to *Mafb*-regulated Cre recombinase expression and recombination in floxed alleles

**Figure 3:**
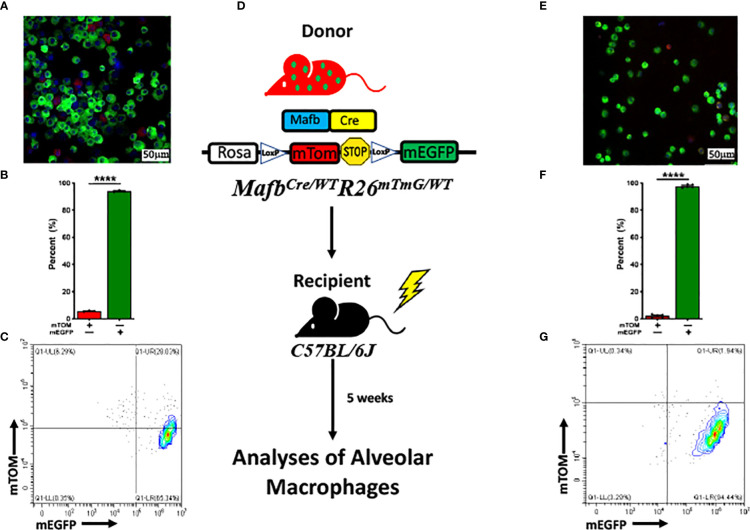
*Mafb^Cre/WT^R26^mTmG/WT^* bone marrow-derived macrophages (BMDMs) are highly amenable to Cre-LoxP recombination *in vitro* and *in vivo*. **(A)** Representative fluorescent photomicrograph depicting the fluorescent cell composition, **(B)** respective percentage of exclusively mTOM+ and mEGFP+ cells. The experiment was repeated three times. Error bars represent SEM ****p<0.0001 using Student’s *t* test. **(C)** Representative flow cytometry graph depicting exclusively mTOM+ (UL) and mEGFP+ (UR+LR) in bone marrow-derived macrophages (BMDMs) cultured from *Mafb^Cre/WT^R26^mTmG/WT^* bone marrow progenitors. **(D)** Generation of chimeric mice *via* transplantation of bone marrow cells from sex- and age-matched *Mafb^Cre/WT^R26^mTmG/WT^* donors to lethally irradiated C57BL/6J recipients. Lung macrophages of chimeric mice were repopulated before subjected to flow cytometry and fluorescent microscopy analyses. **(E)** Representative fluorescent photomicrograph depicting the fluorescent cell composition and **(F)** respective percentage of exclusively mTOM+ and mEGFP+ cells in BALF of chimeric mice. Error bars represent SEM ****p<0.0001 using Student’s *t* test. **(G)** Representative flow cytometry graph depicting exclusively mTOM+ (UL) and mEGFP+ (UR+LR) cells, respectively in alveolar macrophages of chimeric mice (n=5).

Previous report suggests that macrophages under homeostasis are seeded from three separate lineages: yolk sac, fetal liver and bone marrow (38). Therefore, to test whether the concurrent existence of these two alveolar macrophage subpopulations in the lung is dictated by the lung tissue microenvironment or by their differential origins, we analyzed bone marrow-derived macrophages (BMDMs), both *in vitro* as well as *in vivo*, for *Mafb-*regulated induction of mEGFP expression. We generated bone marrow-derived macrophages (BMDMs) using bone marrow progenitors that were harvested from *Mafb^Cre/WT^R26^mTmG/WT^*. Our analyses for the composition of mTOM+ and mEGFP+ BMDMs revealed that ~95% (94 ± 0.4%) of BMDMs were amenable to Cre-LoxP recombination, as indicated by the upregulated mEGFP expression (**Figures 3A–C**). These data suggest that the bone marrow-derived macrophages possess robust *Mafb* expression and *Mafb*-mediated Cre-LoxP recombination.

Since *in vitro*-differentiated BMDMs do not recapitulate the lung tissue microenvironment, further *in vivo* experiments were planned to test whether the bone marrow cells can populate the lung airspaces predominantly with mEGFP+ cells. We hypothesized that bone marrow cells possess robust *Mafb* expression that cause reconstitution of alveolar macrophage population with mEGFP+ cells. Accordingly, 8-week-old C57BL/6 mice were lethally irradiated to deplete cells of hematopoietic lineage, followed by bone marrow transplantation from *Mafb^Cre/WT^R26^mTmG/WT^* age- and sex-matched donor mice. After reconstitution phase, BALF harvested from the chimeric mice were examined (**Figure 3D**). Consistent with the *in vitro* BMDM experiment, ~98% (97.8 ± 0.6%) of the alveolar macrophages were amenable to Cre-LoxP recombination, as indicated by the robust mEGFP expression (**Figures 3E–G**). This data suggest that the majority of alveolar macrophages originated from the bone marrow express *Mafb* gene as compared to fetal liver-derived macrophages.

### Type-2 environment enhances the activity of *Mafb* promoter and promote the *Mafb-*Cre-driven Cre-LoxP recombination

**Figure 4:**
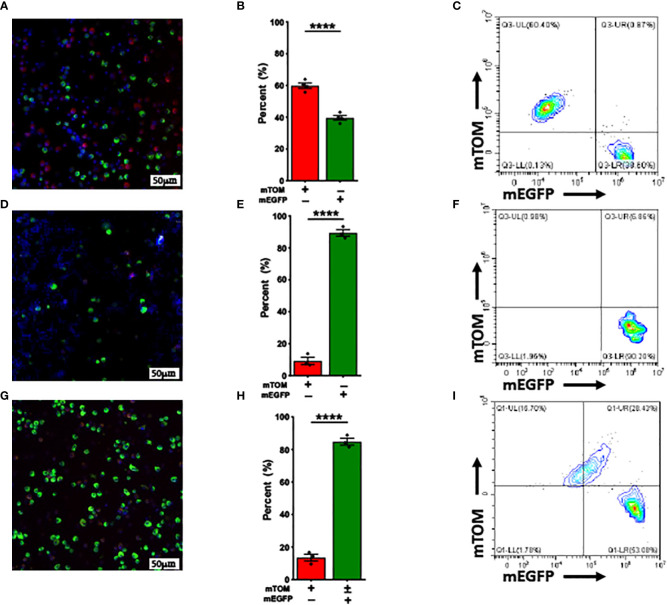
Alternative activation promotes while Classical activation doesn’t affect *Mafb-*Cre-mediated recombination *in vivo.* Representative fluorescent photomicrographs of **(A)** LPS/INF-γ, **(D)** IL-33 and **(G)** ozone-exposed *Mafb^Cre/WT^R26^mTmG/WT^* mice depicting the fluorescent cell composition. Respective percentage of exclusively mTOM+ and mEGFP+ cells in **(B)** LPS/INF-γ, **(E)** IL-33 and **(H)** ozone-exposed *Mafb^Cre/WT^R26^mTmG/WT^* mice. Error bars represent SEM ****p<0.0001 using Student’s *t* test. Representative flow cytometry graphs depicting exclusively mTOM+ (UL) and mEGFP+ (UR+LR), respectively of **(C)** LPS/INF-γ, **(F)** IL-33 and **(I)** ozone-exposed *Mafb^Cre/WT^R26^mTmG/WT^* mice. LPS/IFN-γ-treated mice (n=4), IL-33-treated (n=3) mice, ozone-treated mice (n=3).

*Mafb* gene is known to be upregulated in *Th2* inflammation-associated alternatively-activated (M2) macrophages (39). We further speculated that M2 polarization also promotes *Mafb* promoter upregulation *in vivo*, we challenged adult *Mafb^Cre/WT^R26^mTmG/WT^* mice with LPS [10 μl (10 μg) LPS +40 μl saline]/IFN-γ (40) or IL-33 (1.25 μg IL-33 in 50 μl saline) to induce M1 and M2 activation, respectively. The BALF immune cells collected from these mice were subjected to flow cytometry and fluorescent microscopy analyses to determine the composition of mTOM+ and mEGFP+ macrophages. The Cre-LoxP recombination efficiency, as indicated by the composition of mTOM+ and mEGFP+ macrophages, in LPS/IFN-γ-treated mice (**Figures 4A–C**) was comparable to the naïve *Mafb^Cre/WT^R26^mTmG/WT^* (59.88 ± 1.69% mTOM+ vs 39.63 ± 1.48% mEGFP+). As expected, ~90% (89.4 ± 2.1%) of cells were mEGFP+ in the BALF of IL-33-treated mice (**Figures 4D–F**). These data suggest that M2 polarization promotes Cre-LoxP recombination in macrophages regardless of their origins.

Ozone is known to promote M2 activation in alveolar macrophages (36, 41). Therefore, we hypothesized that ozone exposure will activate *Mafb* promoter in mTOM+ alveolar macrophages of *Mafb^Cre/WT^R26^mTmG/WT^* mice that will facilitate Cre-LoxP-mediated recombination. Therefore, *Mafb^Cre/WT^R26^mTmG/WT^* were repetitively exposed to 800ppb of ozone at 4 hours/day for 14 days and the alveolar macrophages were harvested within 16-24h after the last exposure. BALF cells from these mice were subjected to flow cytometry and fluorescent microscopy analyses. Approximately 85% (84.8 ± 2.1%) of alveolar macrophages collected from ozone-exposed mice lungs expressed mEGFP protein, suggesting that ozone exposure activates *Mafb* promoter in mTOM+ cells that subsequently induces Cre-LoxP-mediated recombination in the reporter allele and thus translates mEGFP protein (**Figures 4G–I** **;** **Supplemental Figure 2**). Approximately 29% of the alveolar macrophages were double positive suggesting continuous transition of mTOM+ into mEGFP+ macrophages (**Figure 4G–I** **;** **Supplemental Figure 2**).

## Discussion

Alveolar macrophages exhibit remarkable plasticity in their response to the extracellular milieu that enables them to perform a variety of functions including maintenance of homoeostasis, immune surveillance, microbial clearance, removal of inhaled biotic/abiotic materials and cellular debris, and resolution of inflammation (42). To elucidate the role of various genes in alveolar macrophage functions, various promoters such as *Lysozyme, Cd11c, Cd11b, Csf1r, Cx3cr1*, and *F4/80*, are commonly employed to induce Cre-LoxP-mediated gene deletion. However, these promoters not only target macrophage but also induce recombination in non-macrophage cell populations. For example, *Lysozyme-*Cre (*LysM-*Cre) targets macrophages, granulocytes, dendritic cells (14), Myeloid-derived suppressor cells (MDSCs) (26), and AT2 cells (23). Similarly, *Csf1r-*Cre targets all leukocyte population (23), *Cd11c-*Cre also targets dendritic cells (29), *Cd11b-*Cre also targets granulocytes, *Cx3cr1-*Cre targets interstitial macrophages (IMs) but not resident alveolar macrophages (23), and *F4/80-*Cre targets only some macrophage subpopulations (24). Therefore, alveolar macrophage-specific promoter amenable to efficient Cre-LoxP-mediated recombination is still awaited.

*Mafb* is a reliable promoter for macrophage lineage tracking in many major organs, such as spleen, small intestine, lung, bone marrow and peritoneal cavity (33). However, whether *Mafb* is specific to alveolar macrophages is not yet known. In this study, we examined *Mafb^Cre/WT^R26^mTmG/WT^* mice where the expression of mTOM/mEGFP (mTmG), a dual reporter floxed allele, was used as a readout for *Mafb*-regulated Cre-LoxP recombination. We hypothesized that *Mafb* promoter activity induces the Cre-LoxP-mediated recombination and the expression of mEGFP protein in alveolar macrophages. To test this hypothesis, first, *Mafb*-regulated recombination efficiency was examined in alveolar macrophages from neonatal versus adult mice. Second, we assessed *Mafb*-regulated recombination efficiency in BMDMs, *in vitro* as well as *in vivo*. Third, the effects of *Th1* versus *Th2* stimuli on *Mafb*-regulated Cre-LoxP-mediated efficiency were compared. Our findings provide interesting insight into the previously unknown association between *Mafb* expression and possibly differential macrophage functionality.

The analyses for *Mafb*-regulated Cre-LoxP-mediated recombination efficiency in steady-state alveolar macrophages from neonatal (PND 3) versus adult (PND 42) mice revealed that ~60% of the harvested alveolar macrophages are not targeted by *Mafb*-regulated Cre recombinase. Further, the lack of double positive (mTOM+ mEGFP+) alveolar macrophages suggest little to negligible ongoing transition of mTOM+ cells into mEGFP+ cells. These data suggest a tightly-regulated distribution of *Mafb-* and *Mafb+* macrophages in steady-state lung airspaces. Tan et al. reported that the fetal liver-derived macrophages enter the alveoli within 1 week after birth and become resident alveolar macrophages (38). Other studies have demonstrated that the bone marrow-derived monocytes contribute to the alveolar macrophage populations (43–45). Our comparison of neonatal and adult BAL macrophages revealed that the dichotomy in the *Mafb* promoter activity is not affected by the early neonatal versus adult age.

Because the *Mafb* expression was restricted to ~40% of the alveolar macrophages, we speculated that the *Mafb* expression pattern might parallel to the expression of other alveolar macrophage-relevant surface markers. The flow cytometry data revealed that both *Mafb*- and *Mafb*+ populations exhibit comparable surface expression patterns for CD11b and CD11c. However, mEGFP+ cells exhibited higher MHCII expression, indicating a more robust antigen presentation potential as compared to mTOM+/mEGFP-cells. *Mafb* expression in macrophages is known to promote their differentiation (46) and maintenance of their M2 phenotype (47, 48). Moreover, *Mafb* gene expression is often accompanied with the upregulation of MHCII expression (49–51). In human, *MAFB* gene was reported to be upregulated in fibrotic lung macrophage clusters of patients with *Th2*-associated diseases such as Idiopathic Pulmonary Fibrosis (IPF) (49), smoking-related lung cancer (52) and SARS-CoV-2 (53). The *Mafb^Cre/WT^R26^mTmG/WT^* mice repetitively exposed to ozone, a known induced of *Th2* inflammation with M2 macrophage predominance (39), exhibited replacement of *Mafb-* alveolar macrophage populations with *Mafb+* macrophages. Interestingly, a double positive mTOM+/mEGFP+ macrophage population with intermediate MHCII expression was identified in ozone-exposed *Mafb^Cre/WT^R26^mTmG/WT^* mice, suggesting that higher MHCII expression is linked to the robust expression of *Mafb* in alveolar macrophages. M2 macrophages possess enhanced antigen presentation ability, thus also express MHCII (54). Therefore, the observed high expression of MHCII in *Mafb+* macrophages was expected. These data suggest that *Th2* tissue environment upregulated the *Mafb* expression and M2 macrophage activation in mice.

Number of reports suggest that tissue macrophages originate from the bone marrow-derived circulating monocytes (55–57) and that the tissue microenvironment, not the lineage, determine the macrophage morphology and function (58). Accordingly, we hypothesized that the higher degree of *Mafb* expression in steady-state alveolar macrophages will be observed in mice that are populated with bone marrow-derived macrophages. Our analyses revealed that the majority of BMDMs *in vitro* are amenable to the *Mafb*-regulated recombination. Consistent with the *in vitro* findings, the *in vivo* model of alveolar macrophages repopulation with BMDMs also revealed *Mafb*-regulated recombination in ~96% alveolar macrophages. These data suggest that the lineage, not the tissue microenvironment, determines the differential expression of *Mafb* in alveolar macrophages.

*Mafb* expression is upregulated in M2 macrophages *in vitro* (39) and is known to promote anti-inflammatory properties in M2 macrophages (59). We reasoned that if *Th2*-predominated milieu upregulates the *Mafb* expression in alveolar macrophages and that, in turn, promotes the Cre-LoxP recombination, the *Mafb-*Cre strain might be useful for Cre-LoxP-mediated recombination in *Th2*-associated studies such as allergic asthma or parasitic diseases. Our data demonstrated that the *Th2* cytokines, indeed, promote the *Mafb-*Cre expression, as indicated by increased number of mEGFP+ macrophages. On the other hand, the *Th1* stimulation did not increase the proportion of mEGFP+ macrophages. These outcomes were comparable between the *in vitro* and *in vivo* stimulation experiments.

Ozone is one of the six criteria environmental pollutants according to the National Ambient Air Quality Standard (NAAQS) (60). The repetitive exposure to ozone results in *Th2*-mediated responses that promote the M2 alveolar macrophages (35, 36, 61–63). Importantly, ozone exposure results in the robust upregulation of *Mafb* transcripts in alveolar macrophages (36). Based on these reports, we hypothesized that the ozone exposure will promote the activation of *Mafb* promoter that, in turn, will induce the Cre-LoxP-mediated recombination in mTmG allele of *Mafb^Cre/WT^R26^mTmG/WT^* mice. As expected, the proportion of mEGFP+ cells increased remarkably and the presence of mTOM+/mEGFP+ cells suggest the induction of *Mafb*-regulated mEGFP expression in the originally mTOM+ cells. Our findings indicate that the presence of *Th2*-predominated responses in the lung airspaces may assist in the high efficiency of *Mafb*-regulated Cre-LoxP-mediated recombination.

The current study has some limitations as well. First, we were not able to examine alveolar macrophages from aged mice. However, we anticipate that the BAL macrophages from aged mice will have greater proportion of mEGFP+ cells. This speculation is consistent with a previous report that suggest age-associated progressive replacement of the embryonically-derived alveolar macrophages with BMDMs (64, 65). Second, we were not able to demonstrate that the mEGFP+ alveolar macrophages are indeed embryonically derived macrophages and were not populated by BMDMs. Although these limitations will be addressed in future studies, the current findings provide a robust foundation for these forthcoming investigations.

In conclusion, this study presents interesting findings: 1) *Mafb* gene expression in alveolar macrophages is lineage-dependent; 2) Bone marrow-derived macrophages exhibit robust *Mafb* expression, *in vitro* as well as *in vivo*; 3) *Th2*, but not *Th1*, environment promotes the activation of *Mafb* promoter, *in vitro* as well as *in vivo*. Finally, this study provides evidence of the coexistence of two macrophage subpopulations, i.e., *Mafb+* and *Mafb-*, in the lung airspaces. While this dichotomy thwarts the use of *Mafb-*Cre in the induction of floxed alleles in alveolar macrophages, this strain provides a unique tool to induce gene deletion in alternatively-activated alveolar macrophages in various *Th2* disease models.

## Data availability statement

The original contributions presented in the study are included in the article/**Supplementary Material**. Further inquiries can be directed to the corresponding author.

## Ethics statement

The animal study was reviewed and approved by Louisiana State University Institutional Animal Care and Use Committee (IACUC).

## Author contributions

TV and YS conceived and designed the study. TV and YS performed all the experiments. TV and YS wrote and reviewed the manuscript for intellectual contents. All authors contributed to the article and approved the submitted version.

## Supplementary material

Supplementary Figure 1
*Mafb-* alveolar macrophages are not precursors of *Mafb+* alveolar macrophages during steady-state. Representative fluorescent photomicrographs of the cultured BAL macrophage cluster from adult naïve *Mafb^Cre/WT^R26^mTmG/WT^* mice (n=3), recorded at day 1, day 2, day 3, day 4, and day 5.

Supplementary Figure 2
*Mafb* upregulation induces expression of MHCII in alveolar macrophages following ozone exposure of *Mafb^Cre/WT^R26^mTmG/WT^* mice. **(A)** Applied Side Scatter (SSC) and Forward Scatter (FSC) to eliminate dead cells and debris. **(B)** Leukocyte CD45+ cells were identified. **(C)** GR1+ cells are identified. **(D)** CD64+ CD24(-) macrophage population is identified **(E)** mTOM+ and mEGFP+ subpopulations were isolated. Myeloid markers such as CD11c, CD11b, MHCII, were assessed in **(F, G)** mTOM+, **(H, I)** mTOM+ mEGFP+ **(J, K)** mEGFP+ subpopulations. **(L)** Histogram depicting mean fluorescence intensity (MFI) of MHCII expression in mTOM+, mTOM+/mEGFP+ and mEGFP+ populations. Error bars represent SEM *****p*<0.0001 using One-way ANOVA. Data shown are from ozone-exposed *Mafb^Cre/WT^R26^mTmG/WT^* mice (n=3).
