# Supplementary figures and images for: Case report: Mafb promoter activity may define the alveolar macrophage dichotomy
Source: Front Immunol. 2022 Dec 12;13:1050494. doi: 10.3389/fimmu.2022.1050494 (PMC9791191; doi:10.3389/fimmu.2022.1050494)

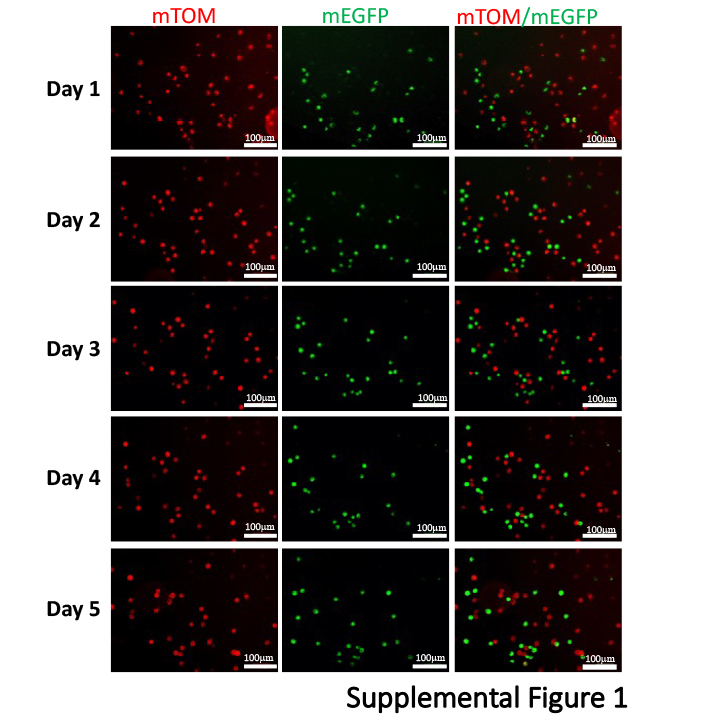

Supplement: Supplementary Figure 1 — Mafb- alveolar macrophages are not precursors of Mafb+ alveolar macrophages during steady-state. Representative fluorescent photomicrographs of the cultured BAL macrophage cluster from adult naïve MafbCre/WTR26mTmG/WT mice (n=3), recorded at day 1, day 2, day 3, day 4, and day 5. [file Image_1.jpeg]

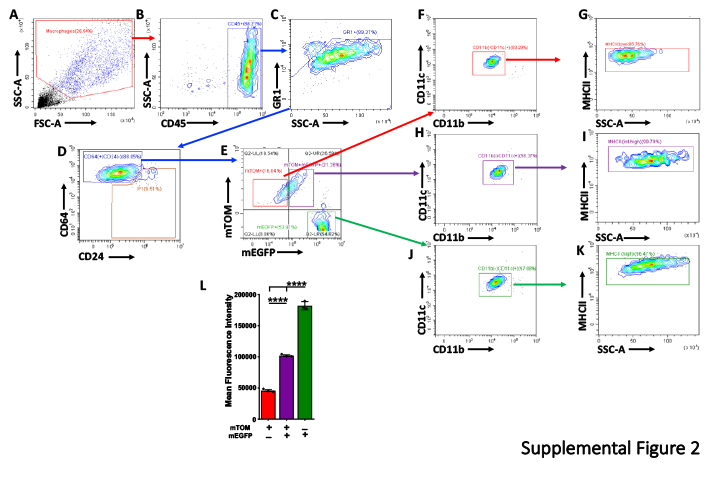

Supplement: Supplementary Figure 2 — Mafb upregulation induces expression of MHCII in alveolar macrophages following ozone exposure of MafbCre/WTR26mTmG/WT mice. (A) Applied Side Scatter (SSC) and Forward Scatter (FSC) to eliminate dead cells and debris. (B) Leukocyte CD45+ cells were identified. (C) GR1+ cells are identified. (D) CD64+ CD24(-) macrophage population is identified (E) mTOM+ and mEGFP+ subpopulations were isolated. Myeloid markers such as CD11c, CD11b, MHCII, were assessed in (F, G) mTOM+, (H, I) mTOM+ mEGFP+ (J, K) mEGFP+ subpopulations. (L) Histogram depicting mean fluorescence intensity (MFI) of MHCII expression in mTOM+, mTOM+/mEGFP+ and mEGFP+ populations. Error bars represent SEM ****p<0.0001 using One-way ANOVA. Data shown are from ozone-exposed MafbCre/WTR26mTmG/WT mice (n=3). [file Image_2.jpeg]
